# Industry 4.0 In-Line AI Quality Control of Plastic Injection Molded Parts

**DOI:** 10.3390/polym14173551

**Published:** 2022-08-29

**Authors:** Saeid Saeidi Aminabadi, Paul Tabatabai, Alexander Steiner, Dieter Paul Gruber, Walter Friesenbichler, Christoph Habersohn, Gerald Berger-Weber

**Affiliations:** 1 Department of Polymer Engineering and Science, Montanuniversitaet Leoben, Otto Gloeckel str. 2, 8700 Leoben, Austria; 2 Polymer Competence Center Leoben GmbH, Rosegger str. 12, 8700 Leoben, Austria; 3 Institute for Production Engineering and Photonic Technologies, Vienna University of Technology, Getreidemarkt 9/311, 1060 Vienna, Austria; 4 Institute of Polymer Processing and Digital Transformation, Johannes Kepler University Linz, Altenberger str. 69, 4040 Linz, Austria

**Keywords:** injection molding of plastics, closed-loop quality control, in-line quality control, AI quality control, predictive control, deep neural network, deep residual learning, surface quality prediction, dimensional features prediction, weight prediction

## Abstract

Automatic in-line process quality control plays a crucial role to enhance production efficiency in the injection molding industry. Industry 4.0 is leading the productivity and efficiency of companies to minimize scrap rates and strive for zero-defect production, especially in the injection molding industry. In this study, a fully automated closed-loop injection molding (IM) setup with a communication platform via OPC UA was built in compliance with Industry 4.0. The setup included fully automated inline measurements, in-line data analysis, and an AI control system to set the new machine parameters via the OPC UA communication protocol. The surface quality of the injection molded parts was rated using the ResNet-18 convolutional neural network, which was trained on data gathered by a heuristic approach. Further, eight different machine learning models for predicting the part quality (weight, surface quality, and dimensional properties) and for predicting sensor data were trained using data from a variety of production information sources, including in-mold sensors, injection molding machine (IMM) sensors, ambient sensors, and inline product quality measurements. These models are the backbone of the AI control system, which is a heuristic model predictive control (MPC) method. This method was applied to find new sets of machine parameters during production to control the specified part quality feature. The control system and predictive models were successfully tested for two groups of quality features: Geometry control and surface quality control. Control parameters were limited to injection speed and holding pressure. Moreover, the geometry control was repeated with mold temperature as an additional control parameter.

## 1. Introduction

The plastics industry is an increasingly demanded market which is known as the third largest manufacturing industry worldwide with a prospective market of 750.1 billion U.S. dollars in 2028 [1]. Most of the products are combined with plastic parts, in which the injection molding manufacturing process contributes approximately 80% of this production in the modern plastics industry [2]. Thus, efficient production in such a big industry is inevitable [3].

Digitized production will dramatically change the value chain of most industrial producers. In Europe, this goal is referred to as Industry 4.0, while in the USA it is focused on by the Industrial Internet Consortium (IIC), in Asia by the Industrial Value-Chain Initiative (IVI), and in China by the initiative “Made in China 2025” [4]. There is an increasing demand for inline and real-time control of the quality of injection molded parts. These requisitions are defining the term ‘injection mold as a cyber-physical system’ in the literature on injection molding technology. This subject designates the requirements for the inline quality feature measurements, machine learning for quality feature prediction, and smart adaptive control of the injection molding process. [5,6,7].

Plastic injection molding (PIM) is a non-linear and very complex process with very dependent process parameters characterizing the quality of the produced parts [8]. Material selection, part geometry, mold design, and process parameters are recognized as the effective parameters for the final quality of the part.

### 1.1. Process Parameters

The quality of the plastic molded part depends on the process parameters in addition to the part design [9]. The subject of which parameters affect the PIM process significantly is the first question to be answered. The significance of process parameters’ effects depends on the expecting quality features. Therefore, a common way is to study the significance of the process parameters by screening before starting a design of experiment (DOE) [10,11,12]. Some researchers select a wide range of process parameters and sensors initially, then, exclude the least significant variables to reduce the number of required experiments [13].

Dimensional properties of the part and the affecting parameters have been one of the most important quality features in PIM [14,15,16,17]. Ferreira Alves [18] and Kapadia [19] provided a comprehensive study on shrinkage and warpage behavior. The history of the most important parameters affecting the shrinkage of the part (or dimensional properties) was studied by Annicchiarico and Alcock [20]. They investigated material properties, processing parameters, and design effects on the shrinkage of the part and concluded the process parameters as the main resource for affecting the shrinkage. The most important process parameters were recognized to be holding pressure, mold temperature, and melt temperature [20]. Recently, many researchers studied shrinkage and warpage of plastic molded parts [19,21,22,23,24,25,26]. In addition, Nurul Hidayah Mohamad Huzaim et al. provide a review on rapid tooling for heat cycle molding [27], while Tim Evens et al. [28] studied the replication of microneedles, in other words, micro-molding. Both technologies can significantly alter the specific effects of injection molding process parameters on dimensional stability. However, in our study, we analyzed a macroscopic part and used conventional mold temperature control.

Selvaraj et al. [29] extracted a list of defects-causes-preventions from the works of literature showing the significant variables over each defect. Among the list, sink marks, weld lines, and flow lines are the relevant defects to this paper. The significant process variables on these defects were reported by Selvaraj et al. as holding pressure, injection speed, and mold temperature.

We consider the weight of the part as a pertinent variable to both other quality features (dimensional properties and surface quality). Therefore, holding pressure time is also considered as affecting the process parameter on the weight of the part.

### 1.2. Process Sensors

The mold as the heart of the injection molding process and part quality provides the most important information about the process. The type and position of the sensors to monitor the process are important topics to be studied. Researchers use in-mold sensors universally [13,30,31]. Florian [32], Ageyeva et al. [33], and Zhao et al. [2] provided comprehensive reviews of the in-mold sensors for injection molding. The multivariable sensor (MVS) is a prevalent sensor in injection molding research. We exerted three MVS sensors in our mold (described in Section 3.3.3) to monitor the behavior of melt during the injection.

Chang et al. [34] propose a real-time online monitoring method to predict the weight of injection molded products made of recycled materials. We used some features of the clamping force since tie-bars elongation is measured as the clamping force by our experiment injection molding machine.

### 1.3. Application of Artificial Intelligence (AI) in PIM

The application of artificial neural networks (ANN) in the injection molding of plastics has been undertaken by many researchers since 1995 [35,36,37]. S. L. Mok et al. reviewed the studies on the process parameters optimization approaches till 1998 [36].

Where support vector machines have been rarely used [38], types of ANNs have been employed widely for the prediction of quality features such as warpage [39], weight [40], shrinkage [41], or cycle time minimization [42].

Ogorodnyk et al. [43] investigated ANN methods and decision trees versus the number of input features. Gim and Rhee [44] employed MLP neural network to predict the weight based on the given features extracted from cavity pressure for part weight optimization. Convolutional neural networks (CNNs) and recurrent neural networks (RNNs) were employed by Nagorny et al. [31] recently. They applied the CNN on thermographic images for part dimension (width) prediction. Despite very few amount of samples (only 177 samples for training and 27 samples for testing), the CNN worked with an accuracy of 87 percent. Guo et al. [45] trained ANN with a reinforcement learning (RL) decision system to optimize the dimensions of an ultrahigh-precision lens product.

We listed the previous modeling and optimization approaches in another study to be support vector machine (SVM), least-square support vector machine (LSSVM), genetic neural fuzzy system (GNFS), fuzzy neural network (FNN), back propagation neural network (BPNN), a combination of genetic algorithms and neural network (GA-NN), deep restricted Boltzmann machine (DRBM), Feedforward neural network (FFNN), MLP, PSO-based BPNN, FFBPNN, hybrid neural network (HNN), Decision trees, CNN, radial basis function (RBF), RL, and Forgetting Factor Recursive Least Square Algorithm (FF-RLS) [4].

Given a review, we conclude that multiple AI solutions may provide an acceptable accuracy of predictions. The application of the AI method type not only depends on the accuracy of the predictions but also on the number of required samples and the time of calculations. Generalizability, transferability, and reversibility of the predicting models are the other important challenges nowadays in this field.

We investigated the applicability of the central composite design, simple regressions, and AI models for plastic injection molding in simulations during a prior study [4] resulting that the ANNs were superior in weight and dimensional properties predictions.

### 1.4. Non-Iterative Optimization Methods

Dang [46] classified the optimization methods into two groups of Meta model-based (for AI methods) and direct discrete methods (for regression), whereas, Zhao et al. [2] categorize the optimization methods into iterative and non-iterative methods. Design of experiment (DOE) as a non-iterative method is used traditionally in research and industry for finding an optimal injection molding process setting in an efficient non-iterative way. In injection molding of plastics, it takes 10 to 50 shots to a stable point of production based on the changes in the machine parameters, and it makes numerous experiments unaffordable [11]. A DOE method is selected based on the complexity of the problem and the affordable amount of experimental production.

The Taguchi method has been used widely by researchers [40,41,47,48,49]. Taguchi’s designs are highly fractionated which makes them very attractive to experimenters [50]. Some researchers use the Taguchi method initially for screening the process variables because of a lower number of experiments [51,52].

Provided that Taguchi is useful to compromise on the number of experiments finding the optimal parameter, the response surface method (RSM) involves more experimentation to get closer to the global optimum. There are two popular classes of RSM designs: central composite design (CCD) and Box-Behnken design (BBD). CCD studies five levels for each experimental variable, which contain two-level factorial or full factorial designs, an additional two axial (star) points for each variable, and a replicated center point [10,50]. Therefore, we have employed a CCD, type inscribed (CCI), in this paper.

### 1.5. Iterative Optimization Methods

Numerous researchers applied iterative optimization methods to find the optimal point of production variables, on which Zhao et al. [2] gave a review. Few of them tried to develop an adaptive online process control strategy. The adaptive control methodologies were conventionally adaptive PID [53] to fuzzy-PID [54,55] and Gray fuzzy PI [56].

Chen and Tung [37] reviewed the history of modeling and closed-loop techniques in 2005. Ogorodnyk [43] provided a general review of the closed-loop control process and AI application for plastic injection molding (PIM) in 2018. Selvaraj et al. [29] presented a comprehensive review of AI applications in PIM recently. We listed the in-line iterative methods for PIM [4] to be Genetic Algorithm (GA), Particle Swarm Optimization (PSO), Invasive Weed Optimization (IWO), Fuzzy Logic, Reverse Neural Network, Model Predictive Control (MPC), and Sequential Approximation Optimization (SAO), and Dynamic Matrix Control (DMC). However, few researchers tried to implement a real-time closed-loop quality control for plastic injection molded parts [55,57,58].

Among all, there are attempts such as Johnston et al. [13] and Hopmann et al. [59] using innovative methods, combining iterative methods with AI models. As a recent example, Tsai et al. [51] developed an adaptive online control system to maintain the consistency of the weight as the quality feature employing BPNNs for prediction.

In this research, we develop a heuristic model predictive control algorithm (MPC) to predict a new set of machine parameters for the desired quality feature in a real-time and in-line way.

### 1.6. OPC UA and Industry 4.0

The key technologies of Industry 4.0, e.g., the Internet of Things, IPv6 and OPC UA, cloud services, big data and artificial intelligence, virtual technologies, and intelligent sensors and actuators are intended to enable manufacturers to remain competitive in the global economy [4]. In November 2016, the Industry 4.0 Platform published a checklist for classifying and advertising products as Industry 4.0 “Basic,” “Ready,” or “Full”. To comply with the “Industry 4.0 communication” criterion, even the lowest category requires the product to be addressable over the network via TCP/UDP or IP and to integrate at least the OPC UA information model [60]. EUROMAP 77 describes the interface between injection molding machines (IMM) and manufacturing execution systems (MES) for data exchange [61]. While OPC UA provides the technology for the transfer of information, EUROMAP 83 defines the definition of which information is transferred and in which form [62].

Therefore, the application of OPC UA platform as an important part of Industry 4.0 was inevitable in our research. We employed three network architectures of the OPC UA platform for three purposes-communication between IMM and stations, sampling the data from IMM and peripherals, and manipulating the closed-loop control. The details of the OPC UA networks are described in Section 2.2.

### 1.7. Research Objective

Our vision is to improve the performance of industrial manufacturing processes for plastics products in terms of product quality as well as process flexibility, efficiency, reliability, and performance through comprehensive process analysis, modeling, control, and digitization. We consider an intelligent injection molding tool to be an indispensable building block in this process: By continuously monitoring the process history in the mold cavity, injection molding machine, and ambient conditions, disturbances can be recognized in an early stage.

The process is modeled using artificial intelligence (AI) for individual quality factors during a training phase. The AI knows the relationships between process parameters, sensor signals, and molded part quality, and uses this knowledge to promptly recognize, evaluate and react adaptively to disturbances combined with a model predictive control (MPC) algorithm to minimize scrap and defective injection molded parts.

## 2. Materials and Methods

### 2.1. Plastics Injection Molding Process

Injection molding of plastics is a cyclic process for manufacturing plastic components. There are different types of injection molding processes, however, this process generally includes plasticization (metering), injection (filling), packing (holding), cooling, and ejecting (demolding). The polymer melts from solid granules through the combination of heat conduction from the heated barrel and heat caused by molecular deformation with the rotation of the screw (shear heating). By closing the mold (clamping), the polymer melt is forced from the barrel (cylinder) into the mold cavity(s) during the injection stage. The molten resin travels through the gate(s), and throughout one or more mold cavities where it will form the desired product(s) shape in the mold. Due to the heat conduction between the mold walls and the melt, the melt temperature decreases, and solidification progresses. Afterward, the cooling stage provides additional time for the resin in the cavity to solidify and become sufficiently rigid for ejection. Finally, the molding machine actuates the necessary cores, slides, and pins to open the mold and eject the molded part(s) [63,64].

### 2.2. OPC UA Communication Platform

Each peripheral device or measurement device comprises an OPC UA server to provide its information and data for the clients. The communication platform is developed based on three main OPC UA clients.

**Figure 1:**
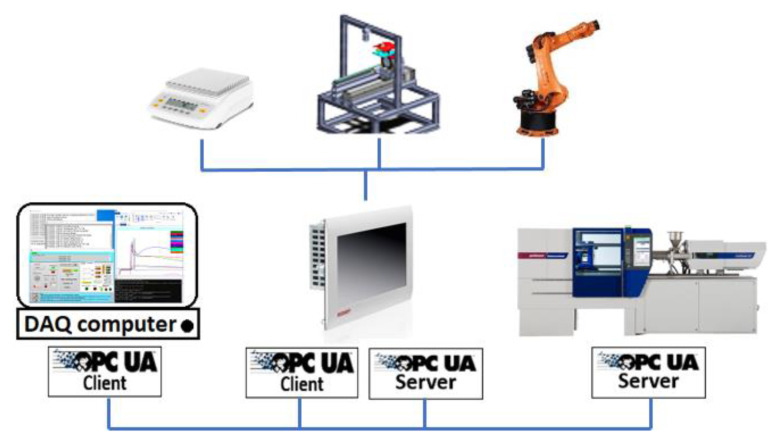
OPC UA platform for management of the measurements and the process automation.

First, A BECKHOFF^®^ IPC model CP6600 (BECKHOFF Automation GmbH, Buers, Germany) is programmed for controlling the axes of the cylindrical measurement system and communications with the other peripheral devices. This IPC is the center of the automation process and communicates with the DAQ center, measurement devices, injection molding machine, and robot. An OPC UA server on this IPC provides the required data (about dimensional measurement status, camera capturing status, and cylindrical moving system status) for communication with the DAQ center (computer). Simultaneously, an OPC UA client on this IPC checks the status of the DAQ center programs and manages the measurements in full compliance with the software on the DAQ center for storing the data. Additionally, this IPC communicates with the robot through digital I/Os (input/output) to guarantee the robot’s safe movements. The OPC UA client on this IPC reads out the status of the injection molding machine, the position of the clamping unit and ejector pins, and the maximum stroke, and manages the permissibility of robot entrance and movements in the machine and mold area. This platform is illustrated in Figure 1.

**Figure 2:**
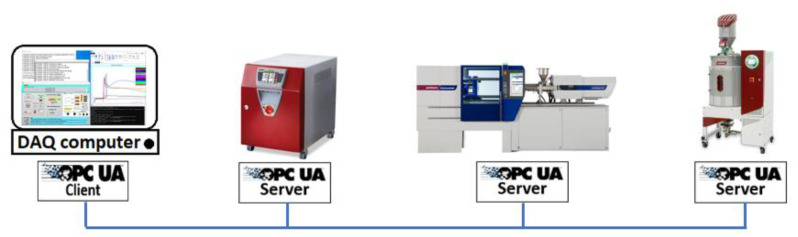
OPC UA communications platform to collect data from injection molding machine and peripherals.

Second, the OPC UA client on the DAQ center is responsible for the acquisition of the injection molding machine data and its peripherals, see Figure 2. The program of this OPC UA client was provided by WITTMANN BATTENFELD GmbH (Kottingbrunn, Austria).

**Figure 3:**
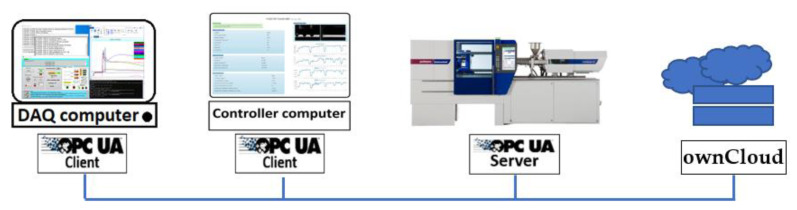
OPC UA communication platform for synchronizing the control process.

Third, the controller synchronizes and applies the outputs of the control process over the OPC UA platform. The DAQ center analyses the sensors and measurements data after each cycle of production. The results of the analyses are uploaded onto the ownCloud, afterward. The controller OPC UA client detects the availability of the data on the ownCloud and after processing the data, the control process takes place. The new set of machine parameters (controlling parameters) is written via controller OPC UA client to the injection molding machine OPC UA server, in case of requirement. The diagram is shown in Figure 3. The entire process of measurement, data analysis, data processing, and control decision-making takes place within one production cycle.

### 2.3. Quality Feedback

In our research, in addition to the weight, dimensional properties, and surface quality are considered to be the crucial quality features for in-line quality control.

#### 2.3.1. Weight

**Figure 4:**
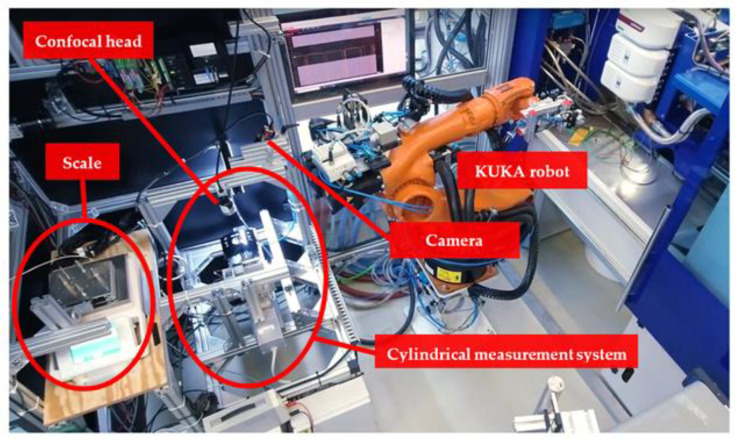
The scale for measuring the weight, the dimensional measurement system, and the camera for surface quality inspection.

A Sartorius scale model Sartorius MSA 2203S-100-DR (Sartorius Lab Instruments GmbH & Co. KG, Goettingen, Germany) measures the weight of the parts with an accuracy of 0.001 g (see Figure 4). A software written in LABVIEW (scale software) communicates with the scale over the RS232 serial protocol. Scale software manages the measurement process and communications with the robot for placing and picking the part. A fixture is positioned on the scale to guarantee a repeatable position for the part during the robot entry. Since the scale is very sensitive to air movements in the measurement room, the scale software manages the stability of the scale, before and after part placement.

#### 2.3.2. Dimensional Properties

**Figure 5:**
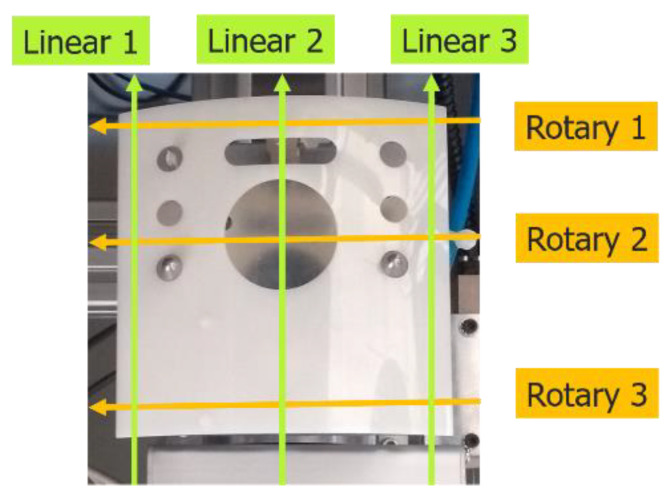
The position of the six scanning lines.

A special three-dimensional cylindrical measurement system was built to measure the dimensional properties of the molded part (illustrated in Figure 4) with ±0.005 mm precision. Details of this measurement system were published in our paper [5] recently. Due to in-line and real-time measurements (a complete measurement should be finished within a production cycle), only the lengths of three linear and three rotary profiles of the molded part were measured (see Figure 5). Later, the controller makes an average of each three-line group for simplicity of the control process.

#### 2.3.3. Surface Inspection

For the aspect of surface inspection, extensive experiences could be drawn upon. In several previous research projects, inspection methods for complex 3D components were developed at the Polymer Component Center Leoben GmbH (PCCL) [65,66,67]. These include methods that can cope with very difficult reflective or highly reflective surfaces [68,69,70].

In our research, a monochrome vision camera (AVT Mako U-130B) was installed at an angle of about 45° to the horizon looking to the center of rotations. An LED-bar light source with a length of 20 cm illuminated the center of the part under the camera (shown in Figure 4). The camera recorded 250~300 images during both rotations for a complete surface scan of the part.

The captured pictures from the surface of the part were analyzed and quantified for three groups of surface defects: flow line thickness, streaks, and sink marks. For this purpose, an offline model-based supervised learning method was developed using the produced samples from the preliminary experiments [71]. The levels of the holding pressure were defined as the numeric classes (labels) and a multi-class classifier based on ResNet-18 [72] was trained. To transform the pressure-level classification result of a model into continuous numerical results, the following equation was applied:

(1) $$Q_{S}=(C_{P}-\min(C_{P}))/\left(\max\left(C_{P}\right)-\min\left(C_{P}\right)\right),$$

where $Q_{S}$ is the surface quality value defined between 0 and 1, and $C_{P}$ is the class of the pressure level. It’s defined so that a higher value of $Q_{S}$ indicates good quality and a lower value means a surface quality containing large effects of flow lines, streaks, and sink marks.

### 2.4. Process Automation

**Figure 6:**
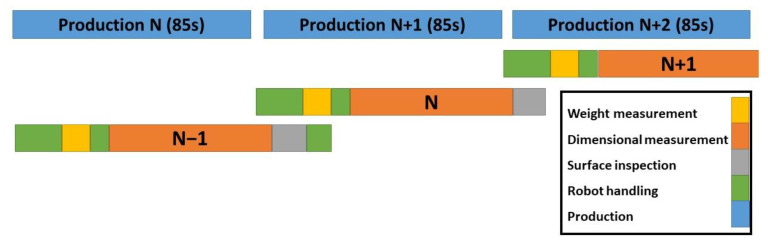
Automation process sequence diagram.

**Figure 7:**
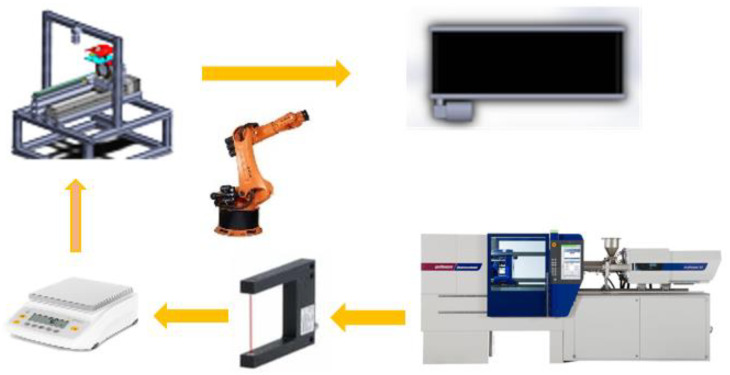
The procedure of handling and measurement of the injection molded part [5].

A KUKA robot (KUKA CEE GmbH, Steyregg, Austria) was programmed to handle the part between the measurement stations. After taking the part from the open injection mold, the part was tested initially for a possible rotation during ejection using a fork sensor, then placed on the scale for weight measurement. Afterward, the robot handles the part to the cylindrical measurement system for dimensional measurements and surface inspection. The sequence diagram is shown in Figure 6 and the procedure is illustrated in Figure 7.

Since the time consistency of the production is important, the part manipulations and measurements should be accomplished during one cycle of injection molding production. The table of robot station actions and parallel movements is given in Table 1 (also see Figure 6).

### 2.5. AI Models

The process was modeled for two groups of outputs. The first group is the quality features including surface quality, weight, and dimensional properties, which consist of the length of 6 profiles of the part. The second group is the sensor data including the digital sensor data and analog sensor data.

Therefore, two groups of models were trained. The first group includes five regression ANNs consisting of four hidden linear layers, where the first three layers were activated with a ReLU function. The input layers of ANNs were considered as five machine parameters based on the preliminary experiment (5 inputs), the time difference from the start of production on the same day of the experiment to each sample production (one input), and ambient sensors data (19 inputs). The predicted outputs of each neural network were:

1. Surface quality (single output),
2. Weight (single output),
3. Profile lengths on the part (six outputs),
4. Analog sensor data (163 outputs),
5. Digital sensor data (136 outputs).

The errors of the outputs were calculated by the mean squared error, and the networks were trained using the Adam optimization algorithm. The second group of models was developed based on random forest regression models. Random Forest is a classification and regression algorithm based on the creation of multiple Decision Trees [73]. The inputs for the second group of the models were analog sensor data (163 inputs), the time difference from the start of production on the same day of the experiment to each sample (one input), and the ambient sensors data (19 inputs). Predicted outputs were:

1. Surface quality
2. Weight
3. Six lines’ lengths

### 2.6. Model Predictive Controller (MPC)

**Figure 8:**
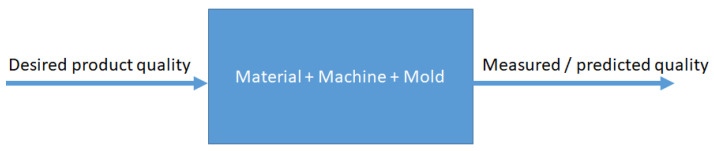
The general block diagram for an injection molding process.

The system in the injection molding process is a combination of an injection molding machine (IMM), material, and mold. The quality of the production is evaluated through measurements, or predictions based on the sensor data acquired during the production (Figure 8).

**Figure 9:**
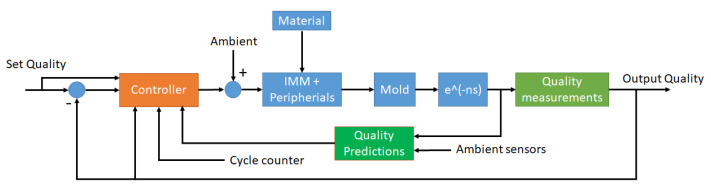
The block diagram of the control system.

**Figure 10:**
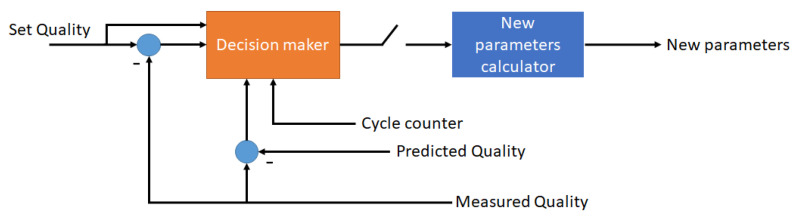
The controller block diagram in detail.

We trained the AI models on the quality features using a preliminary experiment data set and the measurement systems were applied in parallel to investigate the coherence between the predictions and actual measurements. The block diagram of the control system is illustrated in Figure 9. An algorithm decides on a new machine parameters calculations based on the quality error, the difference between the predicted and actual quality values, and the cycle counter. The block diagram of this process is shown in Figure 10.

The control system was built based on the AI models. To simplify the controlling process for the dimensional control problem, the profile lengths were averaged into two groups linear line dimension and rotary line dimension, i.e., the bow length. The designed controller is capable of controlling each of the quality features, “surface quality”, “line dimension”, and “weight”.

#### 2.6.1. Controller Algorithm

A short summary of some of the computational methods is described in our other report [71]. The surface quality, weight, and dimensions of the part are denoted by Q1, Q2, and Q3. as the outputs of the first group of neural network models which were described in Section 2.5. These networks were used to perform a grid-search sub-routine of the controller, described in Algorithm 1.
**Algorithm 1:** Grid search**INPUT**: The current machine parameters *M*, the current machine run time *r*, the vector *E* for ambient sensor data, allowed machine parameters ranges, and a preference function *f* which rates the desirability of a prediction goal and new machine parameters. **OUTPUT**: New machine settings *M’_best_* ***FOR*** each machine parameters vector *M’* so that each element is within the defined range ***DO*** ***|***   Compute the predicted surface quality *s = Q_1_(M’, r, E)* ***|***   Compute the predicted weight *w = Q_2_(M’, r, E)* ***|***   Compute the predicted dimensions *d = Q_3_(M’, r, E)* ***|*   *IF*** *the current loop iteration is the first or if f(s, w, d, M’, M) > f(s_best_, w_best_,*
***|***    *d_best_, M’_best_, M) **THEN*** ***|*   *|***   Set *M’_best_ = M’.* ***|*   *|***   Set *s_best_ = s.* ***|*   *|***   Set *w_best_ = w.* ***|_*    *|_***   Set *d_best_ = d.* ***return*** M’_best_

#### 2.6.2. Extending Grid Search

Since model inference was very fast (depending on the discretization step size), a full enumeration of all possible vectors of the machine parameters was possible. However, specifying certain combinations of the machine parameters reduces the grid search time significantly in cases where the search space is very large. Additionally, some machine parameters (like melt temperature) are known as the slow-changing parameters which require larger n shots between the controller actions to observe the controlling effect significantly in the output quality. Therefore, the controlling parameters were limited to injection speed and holding pressure (and mold temperature in one experiment) in the experiments of this research.

#### 2.6.3. Controller Confidence Scaling

We denote the neural network regressions by S_1_ and S_2_ for predictions of analog and digital sensor data, respectively, with the same input data as the models Q_1_, Q_2_, and Q_3_. Then, the confidence of the machine parameters prediction could be defined based on the accuracy of S_1_ and S_2_ (analog and digital sensor data) predictions. The result could be applied as a proportional delta value to scale the controller’s suggested machine parameters. The MPC controller is described in Algorithm 2.
**Algorithm 2**: Controller**INPUT**: Quality threshold for the quality goal (surface quality, weight, or dimensions), integer *n* for the minimum shot distance between the controller actions, and the reference function *f* as described in Algorithm 1. **OUTPUT**: No output, the algorithm runs perpetually. ***WHILE***
*forever **DO*** ***|  Part Production*** *|  |_  * A new part is produced ***|  Quality Measurement*** *|*  *|  * The surface quality s_measured_ is inspected using CNN. *|*  *|*   The weight w_measured_ is measured using the scale. *|*  *|_*  The dimensions d_measured_ are measured using the cylindrical dimension *|*     measurement system. ***|*  *Quality Prediction*** *|*   *|*   Let *M* be the vector of current machine parameters, *r* the running time of *|*   *|*   the machine, *E* the vector of current ambient sensor data, and *A* the vector *|*   *|*   of current analog sensor data. *|*   *|*   The surface quality *s_pred_* is predicted by *R_1_(M, r, E, A)*. *|*   *|*   The weight *w_pred_* is predicted by *R_2_(M, r, E, A)*. *|*   *|_*  The dimensions *d_pred_* are predicted by *R_3_(M, r, E, A)*. ***|*  *Quality Control*** *|*   *|*   ***IF*** *the measured quality value is outside of the given threshold* ***THEN*** *|*   *|*  *|*
**IF** *there has been a machine parameters update within the last n shots **THEN*** *|*   *|*  *| |_* Terminate this loop *|*   * |*  *|***IF**
*the measured quality deviates strongly from the predicted quality **THEN*** *|*   *|*  *| |_* Warn the user: an external factor might influence the part quality. *|*   *|*  *|_*Use *Algorithm 1* to calculate the new machine parameters based on *M,* *|*   *|*    *r, E,* and *f*. *|_*    *|_*   *|_*Set the new machine parameters.

## 3. Experimental Setup

### 3.1. Experiment Devices and Material

**Figure 11:**
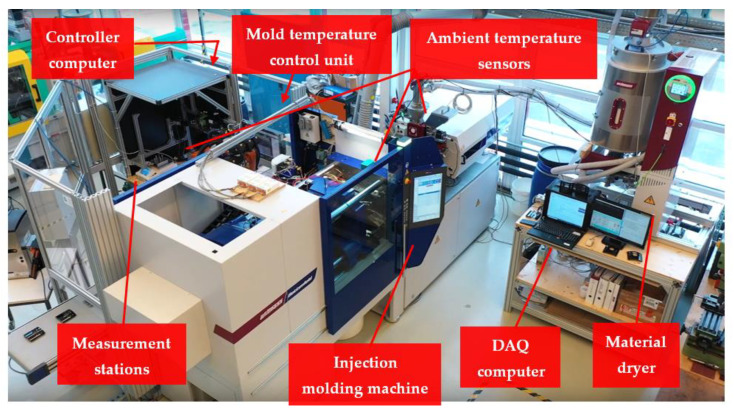
Experimental setup.

Standard white ABS material (acrylonitrile butadiene styrene) Novodur HH-112 from INEOS Styrolution is selected as a popular material in the injection molding industry. Masterbatch Renol-black CAV 80036 from Clariant Masterbatches GmbH was added by DOSIMAX color dosing unit with a ratio of 4% of the metering material into the hopper end. An EcoPower 110 injection molding machine with a screw size of 35 mm from WITTMANN BATTENFELD GmbH was used to process the material. The mold temperature was controlled by a combination of two TEMPRO plus D180 and D90 for fast and wide-ranging mold temperature control (between 80 and 110 degrees Celsius). A KUKA robot KR 5 arc was programmed for handling the molded part between the injection molding machine and stations. The setup is illustrated in Figure 11.

### 3.2. Case Study

**Figure 12:**
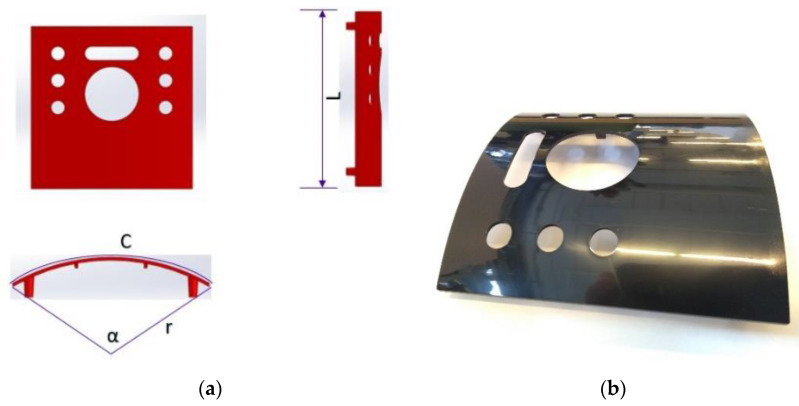
The illustration of the measuring sample, (**a**) Isometric view of the sample, (**b**) The highly reflective surface of the sample is called a piano-black surface.

The part is a highly reflective and partially cylindrical-shaped sample with the cavity dimensions of L = 120.2 mm, outer radius r = 123 mm, curve (bow) length C = 125.36 mm, and arc angle α = 58.39 °, as illustrated in Figure 12. The post-molded volumetric shrinkage ratio for ABS (Acrylonitrile Butadiene Styrene) is expected to be 0.4 to 0.7%. The aggregation of black color, very low surface roughness, and cylindrical shape cause the problem as the hard example for surface inspection and surface quality control.

### 3.3. Process Sensors

#### 3.3.1. Digital Sensor Data

The data from the injection molding machine, DRYMAX dryer, and TEMPRO plus devices were sampled over the OPC UA network platform. The injection molding machine, DRYMAX dryer, and TEMPRO plus D devices have each an OPC UA server inside which provides the information of the internal sensor and variables to be read or written. A program named DataRetriever provided by WITTMANN BATTENFELD reads the data from the OPC UA servers with a sampling rate of 60 Hz for every cycle of production. These data are called digital sensor data.

A python code extracted a set of 136 features from the digital sensor data. Each extracted feature is a single value that represents a characteristic property of the process. These features were, for instance, maximum, average, integral, difference, time of action, length of action, and time difference properties.

#### 3.3.2. Analog Sensor Data (Machine)

Since a persistent sampling rate of the OPC UA data was limited to 60 Hz, a set of important machine signals such as screw pressure (i.e., pressure in the screw antechamber), screw position, clamping force, and digital signals of the machine process phases were measured using an HBM MX 840B 840B (Hottinger Brüel & Kjaer Austria GmbH, Vienna, Austria) for data acquisition and CATMAN software 840B (version 5.2.1, Hottinger Brüel & Kjaer Austria GmbH, Vienna, Austria) for storing the data with a sampling rate of 600 Hz.

#### 3.3.3. Analog Sensor Data (Mold)

**Figure 13:**
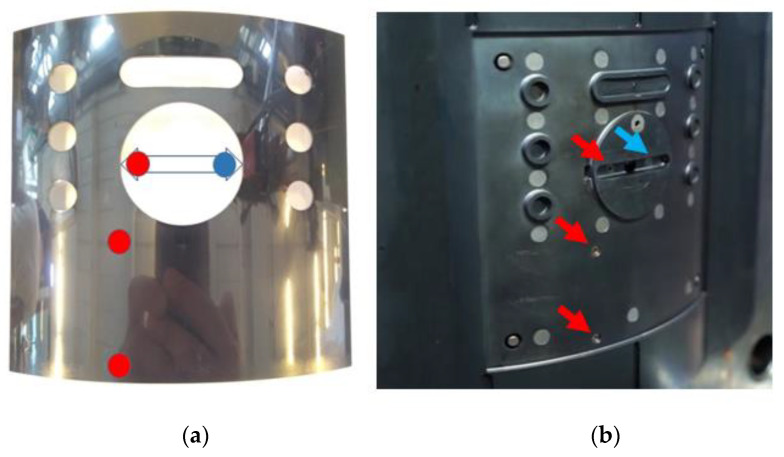
The positions of the FOS P/T sensors are marked in red and the position of the Kistler pressure sensor is marked in blue, (**a**) marked positions on the part, (**b**) marked positions on the moving side of the mold.

Four sensors are installed in the moving side of the mold to monitor the cavity pressure and polymer temperature during the injection, as shown in Figure 13. Three of the sensors were typed on an MTPS 408-IR-BTS-XSR from FOS Messtechnik GmbH (Schacht-Audorf, Germany) and the fourth sensor is a Kistler pressure sensor. MX840B and MX1601 DAQ systems (from HBM) were used for sampling the mold sensor data with a sampling rate of 600 Hz and CATMAN software was used for storing the data.

#### 3.3.4. Ambient Sensors

**Figure 14:**
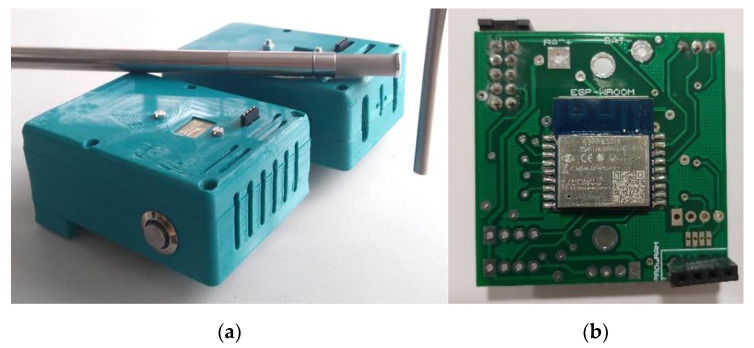
(**a**) Two ambient sensor packages close to an industrial temperature and humidity sensor for calibration, (**b**) The ESP8266 module and sensor board.

Three special ambient sensor packages were built based on the ESP8266 module (Figure 14). Three sensors (BME280, MLX90614, HYT 271) were installed in the ambient sensor package to ensure the safety of the data and the precision of the measurements. Three sensor packages were installed in three zones: room, above mold area, and part quality measurement area. These sensors sample the temperature and relative humidity every 30 s and upload the measurement data every ten minutes onto an ownCloud (developed by Information Center of Montanuniversitaet Leoben, Leoben, Austria).

### 3.4. Design of Experiment (DOE)

A central composite inscribed (CCI) [50] type of CCD was selected to plan the experiments. The levels of the experimental points are shown in Table 2. The combinations of melt temperature, i.e., the set nozzle temperature, and mold temperature, i.e., the set TCU output temperature, were blocked separately since changes in the melt temperature and mold temperature take a long time to reach stability. Therefore, 56 experiments were carried out randomly. To approximate a stable process, a minimum of 40 samples were produced within each experiment. The last 10 shots were used to feed the statistical analysis of the designed DOE, however, all the shots (about 2500 samples) were used for training the AI models to capture the trend of changes in the quality features.

### 3.5. Experiments for Controlling Strategies

The controller was tested with three different controlling strategies. The surface quality was controlled using the injection speed and holding pressure as the controlling parameters. The linear dimension (average of the linear dimensions) was controlled with two different strategies. The first strategy included only injection speed and holding pressure for the controlling parameters. The second strategy included the mold temperature in addition to the first strategy’s controlling parameters.

## 4. Results and Discussion

### 4.1. DOE Results and Factors Correlations

**Figure 15:**
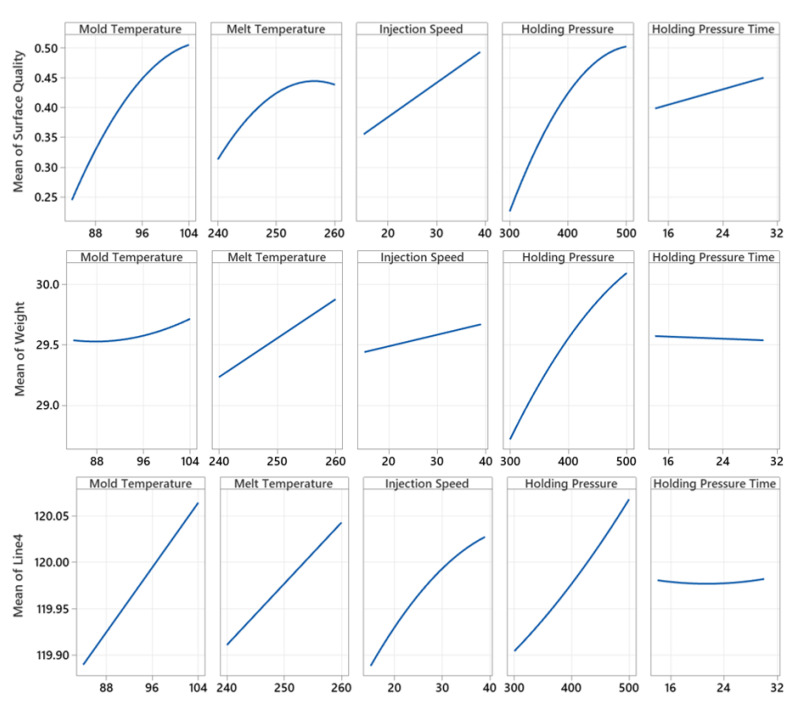
Main effect diagrams from the analysis of the CCI experimental design.

Figure 15 shows the main effect diagrams for weight, surface quality, and Line 4 (as a sample of the line lengths) with the influence of the five investigated experimental factors. The machine parameters, mold temperature, melt temperature, holding pressure, and injection speed, have a significant effect on the quality features.

The R^2^ results of the regression model (using only the DOE factors) for weight were 98.08% and for Line 4 were 97.73% including the block effect in the models, however, the R^2^ value for surface quality response stayed below 70%. A new set of regression models were built using the DOE factors and sensors data. The R^2^ result for Line4 was 98.63% and for weight was 99.26%. The accuracy of the model is not being better for surface quality and the R^2^ remains at about 70%. Models were validated using the K-fold cross-validation method with K = 10.

The precision of the dimensional measurement system was assessed as ±0.010 µm in a previous study [5] and the length of the lines could be predicted with considerable accuracy by regression models.

The main effects agree well with the state of the art when considering a dominant impact of the pin gates and their sealing: Higher mold temperature, higher melt temperature and higher injection speed (via increased shear heating) keep the pin gate longer open to melt flow, thus the holding pressure can be more effective and overwhelms the greater shrinkage potential with lower cooling of the polymer melt, in other words, more polymer is pumped into the cavity with rising mold temperature, melt temperature, and injection speed, making the parts heavier and larger. The minimum holding pressure time of 14 s was chosen to guarantee that the gate is sealed in all experiments within the DOE, thus, as expected, holding pressure time showed no significant influence on dimensions and weight. As predicted in our former simulation study [4], the process parameters prove non-linear influences on the quality outputs. The surface (appearance) quality comprises sink marks, weld-lines, and streaks. Sink marks are shrinkage-dominated surface defects, thus the same dependencies as for dimensions and weight count. Higher contact temperature, higher compression, and lower time for the formation of the frozen layer reduce the strength of weld lines and flow lines, thus rising mold and melt temperatures, and injection speed reduce the visibility of the weld lines at the surface. Allegedly, the replication of (unplanned) micro-scratches in the mirror-finished mold surface by the ABS polymer grows with high mold and melt temperatures, making the part surface rougher, the surface gloss lower and the surface (appearance) quality again worse. A phenomenon that has been reported, for instance in [74,75].

### 4.2. In-Line Closed-Loop Control Results

During each controlling strategy, the machine parameters were changed manually to make disturbances for the controller and investigate the behavior of the controller. These changes were applied to the injection speed and/or holding pressure at some points, and to the mold temperature and melt temperature at some other points.

#### 4.2.1. Surface Quality Control

**Figure 16:**
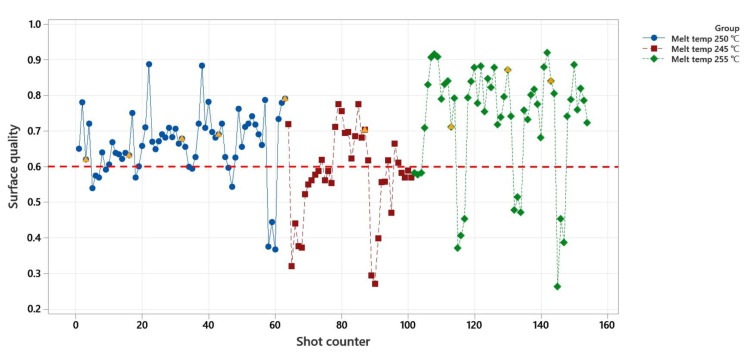
Experiment results for surface quality control. Orange triangles are cycles with manual interference in the process.

The result of the surface quality control is shown in Figure 16. The controller was limited to a distance of three shots between the controlling actions. Additionally, it takes between one to two cycles till the controller can observe the effect of changes on the machine parameters (depending on the time of changes).

The experiment for this control strategy was carried out at three different melt and mold temperatures, respectively 250, 245, and 255 °C melt temperatures and 94, 90, and 98 °C mold temperatures (shown in blue, red, and green in Figure 16). The goal of the controller was to achieve a surface quality value of 0.6 and higher. The injection speed and/or holding pressure were changed respectively to 15 mm/s and 300 bar at the orange triangle-marked shots. The direction of the changes was to decrease the surface quality value.

The result shows that the controller was able to detect the interference after a maximum of two cycles. The controller calculated and applied the new set of machine parameters (injection speed and holding pressure in this control strategy) as soon as it observed the quality reduction. It took a maximum of three cycles for the controller to observe the results of the controlling actions (because one part is under production, one part is on the scale for weight measurement and one part is under the dimensional measurement).

The controller optimized the surface quality for melt temperatures of 250 and 255 °C (along with mold temperatures of 96 and 98 °C) continuously without failure. However, the controller underestimated the necessary controlling action at the melt temperature of 245 °C (along with the mold temperature of 90 °C). This could be addressed by correlations between the surface quality, melt, and mold temperatures.

#### 4.2.2. Linear Dimension Control, Strategy 1

**Figure 17:**
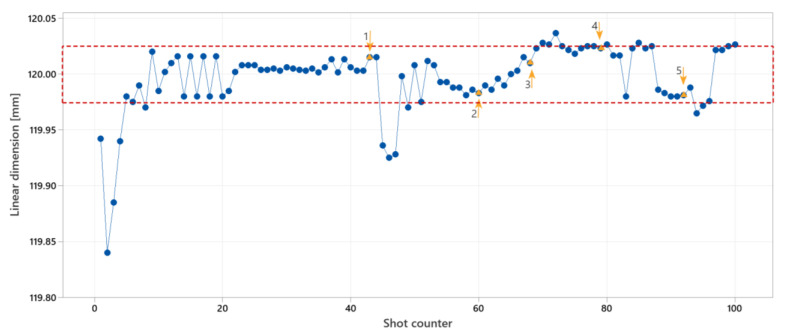
Experiment results for linear dimension control with strategy 1 for controlling parameters. Orange triangles are cycles with manual interference in the process. Arrows show the directions of the change effect.

The result for linear dimension control is given in Figure 17. The controlling goal was a linear dimension of 120 ± 0.025 mm. The tolerance is shown with red dashed lines. The controller was limited to a distance of three cycles between the controlling action. The melt temperature and mold temperature were set to 94 °C and 250 °C respectively.

The manual interference at points 1, 4, and 5 was a decrement of the injection speed and holding pressure respectively to 15 mm/s and 300 bar, and was an increment of the same parameters to 30 mm/s and 450 bar at point 3. The melt temperature and mold temperature were increased respectively to 98 °C and 255 °C at point 2. The dimension of the molded part was increasing cycle by cycle with constant machine parameters. The process control was successful for linear dimension control. However, it seemed that more than three cycles’ distance between the controlling actions is required.

#### 4.2.3. Linear Dimension Control, Strategy 2

**Figure 18:**
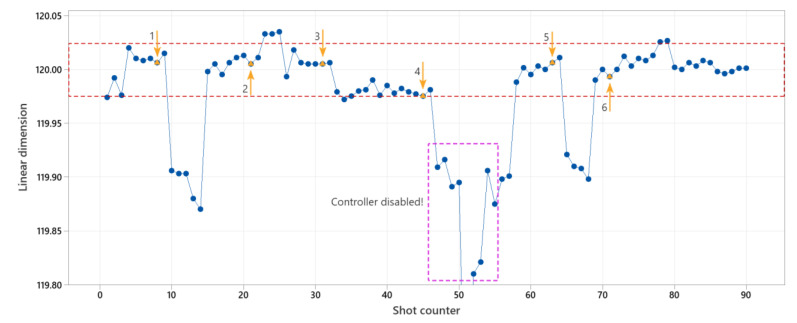
Experiment results for linear dimension control with strategy 2 for controlling parameters. Orange triangles are cycles with manual interference in the process. Arrows show the directions of the change effect.

The controller was tested with the second strategy and the results are shown in Figure 18. Since the mold temperature is a slow variable in the injection molding process, the distance between the controlling actions was changed to ten cycles instead of three.

The manual interference parameters were respectively incensement of injection speed and holding pressure to 15 mm/s and 300 bar at points 1, 4, 5, and additionally decrement of the mold temperature to 90 °C at point 4. The injection speed was decreased to 15 mm/s individually at point 3, and injection speed and holding pressure were respectively increased to 30 mm/s and 450 bar at point 2. The controller was disabled for 9 cycles after point 4 to reset the stable conditions of the controller to initial conditions, and especially observe the first new set point for mold temperature after enabling the controller. At point 6, the melt temperature was increased to 255 °C but the other machine parameters remained unchanged. The controller could successfully predict the appropriate machine parameters (injection speed, holding pressure, and mold temperature) to set the linear dimension within the set tolerance.

#### 4.2.4. Weight Control

Despite that the controller was not tested for weight control, the results of correlations between the weight and other controlling strategies are provided here to illustrate that weight is a more stable and controllable parameter in the injection molding process.

**Figure 19:**
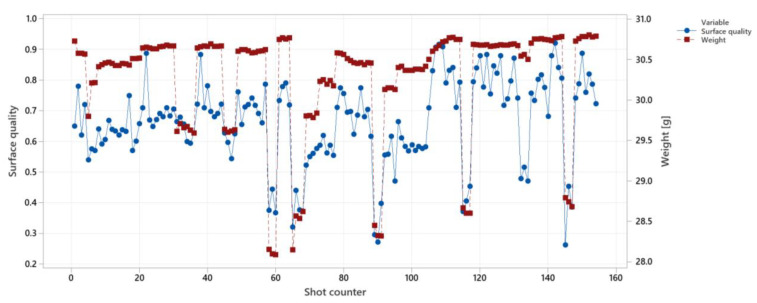
Comparing the correlations between surface quality and weight for surface quality control.

The surface quality and weight are illustrated in Figure 19. As shown, the weight (colored in red) follows the general trend of the changes, since the changing parameters are injection speed, holding pressure, melt temperature, and mold temperature which affect the surface quality and weight in a similar way, see Figure 15. The fluctuations of the weight over the shots are less than for the surface quality.

To compare the fluctuations, we define signal-to-noise ratio (SNR) as shown in Equation (2). Signal-to-noise ratio for the weight in Figure 18 is between 27 to 51 and for the surface quality is between 1.3 to 1.75.

**Figure 20:**
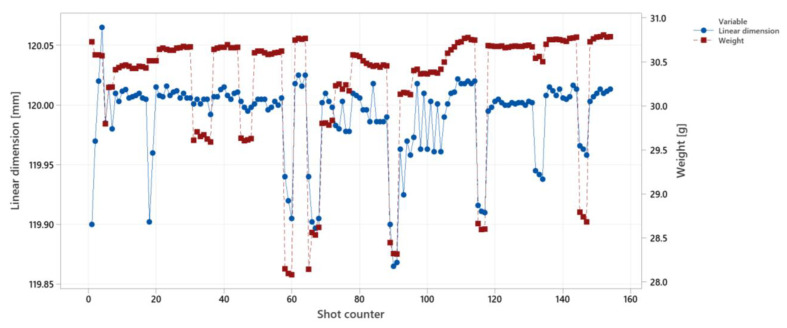
Comparing the correlations between linear dimension and weight for surface quality control.

**Figure 21:**
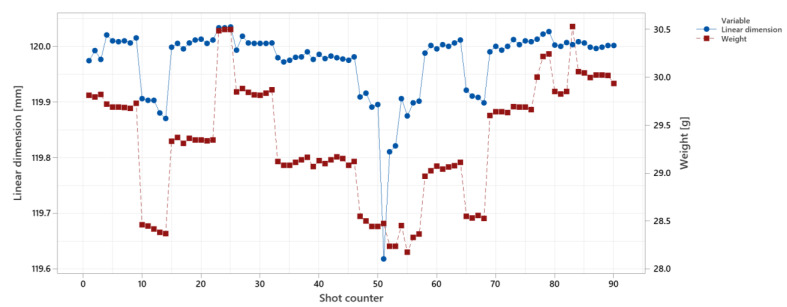
Comparing the correlations between linear dimension and weight for linear dimension control (with strategy 2 for controlling parameters).

The correlations between linear dimension and weight are shown in Figure 20 for the same experiment of the surface quality control. The SNR value for the linear dimensions is between 4 to 9. The same correlation is given for linear dimension control (second strategy) in Figure 21. The trend correlations match for the weight and linear dimension because of similar main factor effects (as shown in Figure 20).

The SNR value of the weight shows that weight has fewer fluctuations and higher resolution rather than surface quality and dimensions against the same variations of the machine parameters. Low fluctuations and high resolutions are two important variables for the controllability of a quality feature.

(2) $$SNR=\frac{Averaged\;controlled\;quality\;value-Averaged\;low\;quality\;value}{Max\;of\;fluctuations-Min\;of\;fluctuations},$$

## 5. Conclusions

In this paper, a novel in-line and fully automated closed-loop process control was presented for the injection molding process. The automation was implemented over the OPC UA network platform in compliance with Industry 4.0 framework for the injection molding process. A novel in-line dimensional measurement system was used to measure the as-molded dimensions of the parts. Therefore, three quality features of the molded parts (weight, dimensional properties, and surface quality) were measured in an in-line and as-molded manner. Initially, a set of experiments through a CCI type DOE were planned to train eight types of AI models. Afterward, the models were applied along with a predictive controller to control the injection molding process for a quality feature. The controller was tested with three different control strategies for the control goal. The surface quality was controlled to achieve a surface quality value higher than 0.6 (where 1 is the best surface quality). The linear dimension (the average of three measured linear dimension lines) was controlled for the goal of 120 mm with a tolerance of ±0.025 mm. The quality features were predicted through the AI models and applied to make the control decision in comparison with the in-line measurements. The controller could successfully control the process for all the strategies. Since injection molding process is a discrete and slow process, the distance between the controlling actions appears to be important in preventing fluctuations.

It would be possible to exclude the in-line measurements after training the AI models, in future research. The process is influenced by many parameters including the environmental variables. Monitoring the trend of the changes could be combined with the control strategy to make decisions for the suitable cycle of control action. Multi-objective process control is the next stage of this research to investigate the possibility of quality control in multiple aspects.

## Figures and Tables

**Table 1:** The time duration for each automation station and parallel tasks.

Action	Duration	Unit	Parallel Actions
Dimensional measurement	44	Second	Robot handling and weight measurement
Camera scan	9	Second	Robot handling and weight measurement
Weight measurement	8	Second	Robot handling
Robot handling	42	Second	Measurements and part production
Production of the part	76~95	Second	A complete measurement process

**Table 2:** Factor levels in designing the CCI experiments.

Factor	Unit	−1	−0.42	0	+0.42	+1
Melt temperature	°C	240	246	250	254	260
Holding pressure	bar	300	358	400	442	500
Time of holding pressure	s	14	18	22	26	30
Mold temperature	°C	84	90	94	98	104
Injection speed	mm/s	15	22	27	32	39

## Data Availability

The data presented in this study are available upon request from the corresponding authors.
